# Associations of resistance training levels with low muscle mass: a nationwide cross-sectional study in Korea

**DOI:** 10.1186/s11556-024-00339-6

**Published:** 2024-03-07

**Authors:** Jae Ho Park, Nam-Kyoo Lim, Hyun-Young Park

**Affiliations:** 1 Division of Population Health Research, Department of Precision Medicine, Korea National Institute of Health, Korea Disease Control and Prevention Agency, 200 Osongsaengmyeong2-ro, Osong-eup, Heungdeok-gu, Cheongju-si, Chungcheongbuk-do 28160 Korea; 2 Korea National Institute of Health, Korea Disease Control and Prevention Agency, 187 Osongsaengmyeong2-ro, Osong-eup, Heungdeok-gu, Cheongju-si, 28159 Chungcheongbuk-do Korea

**Keywords:** Resistance training, Low muscle mass, Fat-free mass index, Population study

## Abstract

**Background:**

Low muscle mass is associated with adverse health outcomes such as functional decline and all-cause mortality. This study investigated the relationship between the risk of low muscle mass and the training period and/or frequency of resistance training (RT).

**Methods:**

We included 126,339 participants (81,263 women) from nationwide cohorts in Korea. Low muscle mass was defined based on the fat-free mass index. To investigate the presence of an inverse dose–response relationship between RT levels and the risk of low muscle mass, the training period (months) and frequency (per week) of RT were used. Multiple logistic regression models were used to assess the risk of low muscle mass according to the RT levels.

**Results:**

Prevalence rates for low muscle mass in our study population were 21.27% and 6.92% in men and women, respectively. When compared with not performing RT, performing RT for 3–4 days/week and ≥5 days/week decreased the risk of low muscle mass by 22% and 27%, respectively, and performing RT for 12–23 months and ≥24 months decreased the risk by 19% and 41%, respectively. When simultaneously considering both training period and frequency, performing RT for either 3–4 days/week or ≥5 days/week was significantly related to risk reduction, provided that the training period was at least 1 year. Importantly, performing RT for more than 2 years resulted in an additional risk reduction. However, there was no additional effect of performing RT for ≥5 days/week compared to 3–4 days/week, regardless of whether the RT duration was 1–2 years or more than 2 years.

**Conclusions:**

Since performing RT for 5 days/week or more did not yield any additional effects on the risk of low muscle mass, performing RT for 3–4 days/week was sufficient to prevent low muscle mass. The effectiveness of this preventive measure can be further enhanced by engaging in long-term RT, specifically for more than 2 years.

**Supplementary Information:**

The online version contains supplementary material available at 10.1186/s11556-024-00339-6.

## Introduction

Since sarcopenia is defined as the age-related reduction in skeletal muscle mass along with the loss of muscular strength and/or reduced physical function [1], low muscle mass is one of the main components for diagnosing sarcopenia. Age-related changes in body composition, including weight loss, increased fat mass, and reduced muscle mass, are common in older adults. It is well established that skeletal muscle mass undergoes a linear decline after the age of 30 years [2]. Moreover, studies have revealed a considerable prevalence of low muscle mass, ranging from approximately 20% to 40%, within the older Asian population [3, 4]. Due to the association between low muscle mass and an elevated risk of physical dependency, osteoporosis, diabetes mellitus, and even all-cause mortality [5–8], there has been a surge of interest in therapeutic and preventive approaches targeting low muscle mass.

Resistance training (RT) refers to a form of leisure-time physical activity (PA) specifically designed to enhance muscular fitness through exercises that involve working muscle groups against external resistance. Recent meta-analytical evidence has shown that regular RT has significant effects on physical and social functioning, muscular strength, muscle mass, and mental health in older adults [9–11]. Thus, the American College of Sports Medicine (ACSM) recommends performing RT for 2–3 days per week for all adults as it is beneficial for maintaining and improving musculoskeletal fitness and overall health [12]. Fortunately, there has been a nearly 10% increase in the proportion of individuals meeting the RT guidelines over the past two decades in the United States [13]. However, despite the growing interest in participating in RT programs, there is a lack of studies investigating the presence of an inverse dose–response relationship between RT volume (e.g., training period and frequency) and the risk of low muscle mass. While a meta-analysis of randomized controlled trials (RCTs) conducted for more than 4 weeks concluded that performing RT twice per week was superior to performing RT once per week in terms of increasing muscle mass [14], further studies are needed to determine whether RT performed at frequencies higher than those recommended by current guidelines provides additional risk reduction for low muscle mass. Moreover, there is a paucity of studies investigating the risk of low muscle mass while simultaneously considering both the training period and frequency of RT.

Therefore, the purpose of the present study was to examine whether an inverse graded dose–response association exists between the training period and frequency of RT and the risk of low muscle mass in Korean adults from large nationwide cohorts. Furthermore, we conducted additional analyses to provide recommendations for preventing low muscle mass by simultaneously considering both the training period and frequency of RT.

## Materials and methods

### Study participants

This study used data from the Korean Genome and Epidemiology Study (KoGES), conducted by the Korea National Institute of Health. The KoGES is a large-scale consortium project consisting of six prospective cohort studies to investigate and assess the genetic and environmental etiologies of non-communicable diseases in Korea, including obesity, hypertension, diabetes mellitus, and cardiovascular diseases [15]. For this study, we used 2003–2013 data from the KoGES_Health Examinee (HEXA) study, including 173,202 urban residents aged 40–79 years, as well as data from the fourth wave of the KoGES_Ansan and Ansung study (2007–2008), including 6,688 participants, aged 44–76 years, who lived in Ansan (an urban area) or Ansung (a rural area). As specific information on RT levels could be retrieved from the fourth wave of the KoGES_Ansan and Ansung study, we included this and not the baseline data. All participants underwent physical examinations and face-to-face surveys conducted by trained medical staff. A detailed description of the KoGES cohort studies has been provided previously [15].

Among the 179,890 participants from the cohorts, 53,551 were excluded from the present study based on the following exclusion criteria: lack of data on fat-free mass (*n* = 44,195), lack of data on leisure-time PA levels (*n* = 4,360), and no data available for the covariates (*n* = 4,996). Overall, 126,339 participants (81,263 women) were included in the final analysis (Additional file 1). This study was approved by the Institutional Review Board Committee of the Korea National Institute of Health, Korea Disease Control and Prevention Agency (Approval No. 2021-04-02-P-A). 

### Measurement of leisure-time PA

All participants completed questionnaires containing details on RT regularity and leisure-time PA levels. RT was defined as any training program involving muscle contraction against external resistance using body weight, weight machines, barbells, or dumbbells. The frequency (per week), training time (min/week), and training period (months) of RT were assessed. Regular RT was defined as participation in an RT program for more than 1 day per week. Participants were classified into two groups based on the regularity of RT: “Non-RT (not performing RT),” and “RT (performing RT).” To investigate the presence of an inverse dose–response relationship between RT levels and the risk of low muscle mass, the training period (months) and frequency (per week) of RT were used. Based on the frequency of RT, participants were categorized into one of five subgroups: “Non-RT (not performing RT),” “1 day/week,” “2 days/week,” “3–4 days/week,” and “≥5 days/week.” Similarly, participants were classified into one of four subgroups based on training period of RT: “Non-RT (not performing RT),” “<12 months,” “12–23 months,” and “≥24 months.”

Regarding leisure-time PA levels, we assessed the intensity, frequency (per week), and duration (min/week) during a typical week. Moderate-intensity leisure-time PA was defined as participating in sports or engaging in exercise that results in sweating. Based on the PA guideline (moderate-intensity leisure-time PA for at least 150 min per week) [16] and RT regularity, participants were categorized into one of four subgroups: “Low-PA (not meeting the guideline),” “Low-PA+RT (not meeting the guideline but performing RT),” “High-PA (meeting the guideline),” and “High-PA+RT (meeting the guideline and performing RT).”

### Definition of low muscle mass

Low muscle mass was defined based on the fat-free mass index (FFMI), which was determined using fat-free mass measured by bioelectrical impedance analysis (BIA) (InBody 3.0, Biospace, Seoul, Korea). The FFMI was calculated by dividing the fat-free mass (kg) by the square of the height (m) (kg/m^2^). According to a recent study on the screening of low muscle mass, the cutoff points of FFMI were 17.5 kg/m^2^ for men and 14.6 kg/m^2^ for women [17].

### Covariates

Our analyses encompassed various sociodemographic and health-related factors, including age, sex, educational level, drinking and smoking habits, PA-time, body mass index (BMI), waist circumference (WC), fat-free mass, blood pressure (BP), hypertension, diabetes mellitus, and laboratory parameters. Educational level was classified as elementary school graduate or lower, middle or high school graduate, and college graduate or higher. Drinking and smoking habits were classified as “never,” “former,” and “current.” PA-time was defined as the total time (min/week) spent engaging in moderate-intensity leisure-time PA.

Anthropometric data, including height, body weight, and WC, were measured by trained healthcare providers using standardized methods. BMI was calculated as body weight (kg) divided by height (m) squared (kg/m^2^). Trained healthcare providers also measured BP using standard protocols. Systolic BP (SBP) and diastolic BP (DBP) were obtained by averaging two readings from the arm with the highest SBP after the participant had rested for 5 min in a seated position. Blood samples were collected after an overnight fasting period of 8 h. Biochemical assays were performed to determine levels of total cholesterol (T-Chol), high-density lipoprotein cholesterol (HDL-C), triglyceride (TG), and fasting blood glucose (FBG). Hypertension was defined based on a previous diagnosis by a physician, current use of antihypertensive drugs, SBP ≥140 mmHg, or DBP ≥90 mmHg. Diabetes mellitus was defined based on a previous diagnosis by a physician, current use of antidiabetic medications, including insulin and oral hypoglycemic agents, FBG ≥126 mg/dL, or glycated hemoglobin ≥6.5%. Detailed information on the biochemical analyses is available elsewhere [15].

### Statistical analysis

All statistical analyses were conducted using SAS software (version 9.4; SAS Institute, Cary, North Carolina, United States). Participant characteristics are presented as descriptive statistics. Continuous variables are presented as mean ± standard deviation, whereas categorical variables are expressed as absolute frequencies and percentages (%). The chi-square test was used to compare educational levels, drinking and smoking habits, RT regularity, and the prevalence of low muscle mass and non-communicable diseases (e.g., hypertension and diabetes mellitus) between the groups. Independent *t*-tests were used to compare age, PA-time, BMI, WC, fat-free mass, FFMI, SBP, DBP, T-Chol, HDL-C, TG, and FBG levels between groups.

A multiple logistic regression model was used to evaluate odds ratios (ORs) and 95% confidence intervals (CIs) for the prevalence of low muscle mass. The models were adjusted for age, sex, drinking, smoking, educational level, BMI, hypertension, and diabetes mellitus. Subgroup analyses were conducted to examine the relationship between RT levels and the risk of low muscle mass, taking into account age (<65 and ≥65 years), sex (male and female), educational level (≤middle school and ≥high school), current drinking habits (no and yes), smoking status (never and ever), BMI (<25 and ≥25 kg/m^2^), hypertension (no and yes), and diabetes mellitus (no and yes). The *p*-value for the interaction was estimated to assess the consistency of the associations across the subgroups. All tests were two-tailed, and statistical significance was set at a *p*-value < 0.05.

## Results

Table 1 presents the characteristics of the study participants. The prevalence rates of low muscle mass were 21.27% in men and 6.92% in women. Men had a higher mean age compared to women. The prevalence rates of a high educational level (≥college), current drinking and smoking, RT regularity, hypertension, and diabetes mellitus were higher in men than in women. In terms of other variables, men had markedly higher values for PA-time, BMI, WC, fat-free mass, FFMI, SBP, DBP, TG, and FBG, while having lower levels of T-Chol and HDL-C, as compared to women.

**Table 1:** Characteristics of study participants

**Variables**	**Men**	**Women**	***p*-value**
	**( *n* = 45,076)**	** (*n*** **= 81,263)**	
**Age** (years)	54.09 ± 8.80	52.99 ± 8.18	< 0.0001
**Educational level**, n (%)			< 0.0001
*≤Elementary school*	4,288 (9.51)	16,611 (20.44)	
*Middle/high school*	24,201 (53.69)	48,770 (60.02)	
*≥College*	16,587 (36.80)	15,882 (19.54)	
**Drinking habit**, n (%)			< 0.0001
*Never drinker*	9,034 (20.04)	54,908 (67.57)	
*Ex-drinker*	3,219 (7.14)	1,466 (1.80)	
*Current drinker*	32,823 (72.82)	24,889 (30.63)	
**Smoking habit**, n (%)			< 0.0001
*Never smoker*	11,987 (26.59)	78,243 (96.28)	
*Ex-smoker*	18,402 (40.83)	1,037 (1.28)	
*Current smoker*	14,687 (32.58)	1,983 (2.44)	
**PA-time** (min/week)	183.69 ± 262.32	145.83 ± 220.11	< 0.0001
**RT**, n (%)	6,380 (14.15)	7,740 (9.52)	< 0.0001
**BMI** (kg/m^2^)	24.38 ± 2.77	23.68 ± 2.99	< 0.0001
**WC** (cm)	85.71 ± 7.60	78.62 ± 8.39	< 0.0001
**Fat-free mass** (kg)	53.08 ± 5.75	39.77 ± 3.99	< 0.0001
**FFMI** (kg/m^2^)	18.62 ± 1.49	16.26 ± 1.26	< 0.0001
**Low muscle mass**, n (%)	9,586 (21.27)	5,622 (6.92)	< 0.0001
**SBP** (mmHg)	124.89 ± 14.40	120.53 ± 15.14	< 0.0001
**DBP** (mmHg)	78.39 ± 9.73	74.60 ± 9.66	< 0.0001
**T-Chol** (mg/dL)	192.56 ± 34.84	199.02 ± 35.60	< 0.0001
**HDL-C** (mg/dL)	48.70 ± 11.86	55.37 ± 12.95	< 0.0001
**TG** (mg/dL)	150.41 ± 107.42	113.98 ± 74.38	< 0.0001
**FBG** (mg/dL)	99.41 ± 24.42	93.12 ± 19.40	< 0.0001
**Hypertension**, n (%)	15,885 (35.24)	21,126 (26.00)	< 0.0001
**Diabetes mellitus**, n (%)	6,090 (13.51)	6,530 (8.04)	< 0.0001

*PA-time* total time of regular participation in any sport or exercise to the point of sweating, *RT* resistance training, *BMI* body mass index, *WC* waist circumference, *FFMI* fat-free mass index, *SBP* systolic blood pressure, *DBP* diastolic blood pressure, *T-Chol* total cholesterol, *HDL-C* high-density lipoprotein cholesterol, *TG* triglycerides, *FBG* fasting blood glucose

The characteristics of the study participants based on RT regularity and sex are shown in Additional file 2. The proportion of individuals engaging in regular RT in our study population was 14.15% among men and 9.52% among women. The prevalence of low muscle mass was significantly higher in the Non-RT group than in the RT group, irrespective of sexes. In both men and women, the RT group exhibited a significantly lower mean age, WC, TG, FBG, and a lower proportion of never drinkers, current smokers, and patients with diabetes mellitus in comparison to the Non-RT group. Conversely, the RT group demonstrated higher PA-time, fat-free mass, HDL-C, and a higher prevalence of a high educational level (≥college) compared to the Non-RT group. In men, the RT group was significantly associated with higher BMI, FFMI, SBP, and DBP, whereas in women, the RT group showed lower BMI, FFMI, SBP, DBP, T-Chol, and a lower prevalence of hypertension, as compared to the Non-RT group.

**Fig. 1:**
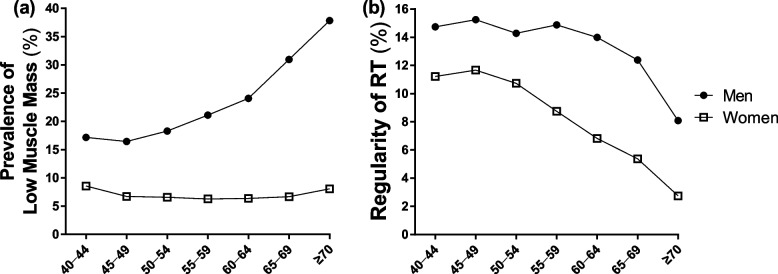
Comparing (**a**) low muscle mass prevalence and (**b**) resistance training regularity by age and sex. RT, resistance training

Figure 1 illustrates the comparison of prevalence of low muscle mass and RT regularity according to sex and age. In our study population, there was a steady increase in the prevalence of low muscle mass, particularly in men, but not in women. There was a significant decrease in the proportion of individuals engaging in regular RT among men aged ≥65 years. Conversely, among women, there was a steady decrease in the proportion of regular RT with advancing age.

An inverse association between RT regularity and the risk of low muscle mass was observed after adjusting for covariates (Additional file 3). Performing RT was associated with a significant decrease in the risk of low muscle mass in 22% of both men and women (all *p* < 0.0001). We further analyzed the additional effects of RT on low muscle mass in participants with high leisure-time PA levels (Additional file 4). The results indicated that incorporating RT into the routine of participants with high leisure-time PA levels further reduced the risk by 24% in both men and women (all *p* < 0.001).

We conducted further investigations to explore the presence of an inverse dose–response association between the training period, frequency of RT, and the risk of low muscle mass. As shown in Table 2, among men, performing RT for 3–4 days/week and ≥5 days/week was associated with a risk reduction of 24% (*p* < 0.001) and 27% (*p* < 0.001), respectively, in comparison to not performing RT (*p* for trend < 0.0001). Among women, compared to those in the Non-RT group, performing RT for 3–4 days/week and ≥5 days/week led to a risk reduction of 20% (*p* < 0.05) and 27% (*p* < 0.01), respectively (*p* for trend < 0.0001). While the total RT time per week was significantly higher in the ≥5 days/week group than in the 3–4 days/week group (all *p* < 0.0001) for both sexes, there was no significant difference in the risk of low muscle mass between these groups. In addition, as presented in Table 3, among men, compared to those in the Non-RT group, performing RT for 12–23 months and ≥24 months was associated with a risk reduction of 20% (*p* < 0.001) and 43% (*p* < 0.0001), respectively (*p* for trend < 0.0001). Among women, compared to those in the Non-RT group, performing RT for 12–23 months and ≥24 months was related to a risk reduction of 18% (*p* < 0.01) and 40% (*p* < 0.01), respectively (*p* for trend < 0.0001).

**Table 2:** Odds ratios for low muscle mass prevalence according to RT frequency and sex

	**n**	**RT levels**				**Crude model** OR (95% CI)	**Adjusted model** OR (95% CI)
		**Frequency**	**Time**	**Training period**			
		(days/week)	(min/week)	(months)	≥1 year (%)		
**Total**							
*Non-RT*	112,219	0.00 ± 0.00	0.00 ± 0.00	0.00 ± 0.00	0.00	1 (reference)^a^	1 (reference)^a^
*1 day/week*	782	1.00 ± 0.00	44.51 ± 31.59	18.08 ± 41.51	70.84	1.30 (1.07–1.58)^**^	0.98 (0.74–1.32)
*2 days/week*	1,859	2.00 ± 0.00	103.86 ± 59.89	27.95 ± 54.47	79.29	0.92 (0.80–1.07)	0.84 (0.69–1.03)
*3–4 days/week*	6,329	3.42 ± 0.49	201.98 ± 116.28	25.24 ± 46.85	82.04	0.75 (0.69–0.82)^****^	0.78 (0.69–0.88)^****^
*≥5 days/week*	5,150	5.86 ± 0.89	368.36 ± 214.38	24.76 ± 47.87	87.65	0.87 (0.80–0.95)^**^	0.73 (0.64–0.83)^****^
**Men**							
*Non-RT*	38,696	0.00 ± 0.00	0.00 ± 0.00	0.00 ± 0.00	0.00	1 (reference)^a^	1 (reference)^a^
*1 day/week*	394	1.00 ± 0.00	43.66 ± 31.87	22.65 ± 54.80	73.10	1.00 (0.79–1.27)	1.17 (0.80–1.71)
*2 days/week*	849	2.00 ± 0.00	101.08 ± 61.70	32.48 ± 66.85	81.15	0.73 (0.61–0.87)^***^	0.85 (0.65–1.12)
*3–4 days/week*	2,673	3.44 ± 0.50	193.62 ± 121.23	28.69 ± 56.82	84.55	0.62 (0.56–0.69)^****^	0.76 (0.65–0.89)^***^
*≥5 days/week*	2,464	6.01 ± 0.91	350.32 ± 222.82	27.29 ± 56.71	90.38	0.74 (0.66–0.82)^****^	0.73 (0.62–0.86)^***^
**Women**							
*Non-RT*	73,523	0.00 ± 0.00	0.00 ± 0.00	0.00 ± 0.00	0.00	1 (reference)^a^	1 (reference)^a^
*1 day/week*	388	1.00 ± 0.00	45.38 ± 31.32	13.44 ± 19.62	68.56	1.22 (0.86–1.75)	0.74 (0.46–1.19)
*2 days/week*	1,010	2.00 ± 0.00	106.20 ± 58.26	24.14 ± 40.94	77.72	0.94 (0.73–1.20)	0.81 (0.60–1.11)
*3–4 days/week*	3,656	3.40 ± 0.49	208.09 ± 112.15	22.72 ± 37.76	80.20	0.75 (0.65–0.87)^***^	0.80 (0.67–0.96)^*^
*≥5 days/week*	2,686	5.71 ± 0.85	384.91 ± 204.98	22.44 ± 37.86	85.15	0.69 (0.58–0.83)^****^	0.73 (0.59–0.91)^**^

*RT* resistance training, *OR* odds ratio, *CI* confidence interval, *BMI* body mass index

^a^*p* < 0.0001 in the test for trend of ORs, ^*^ *p* < 0.05, ^**^ *p* < 0.01, ^***^ *p* < 0.001, ^****^ *p* < 0.0001. Adjusted for age, sex, drinking, smoking, educational level, BMI, hypertension, and diabetes mellitus

**Table 3:** Odds ratios for low muscle mass prevalence according to training period of RT and sex

	**n**	**RT levels**			**Crude model** OR (95% CI)	**Adjusted model** OR (95% CI)
		**Frequency**	**Time**	**Training period**		
		(days/week)	(min/week)	(months)		
**Total**						
*Non-RT*	112,219	0.00 ± 0.00	0.00 ± 0.00	0.00 ± 0.00	1 (reference) ^a^	1 (reference) ^a^
*<12 months*	2,386	3.52 ± 1.56	206.23 ± 170.38	4.94 ± 2.95	0.85 (0.75–0.97)^*^	0.86 (0.72–1.04)
*12–23 months*	9,496	4.12 ± 1.72	248.54 ± 191.56	12.04 ± 0.47	0.90 (0.84–0.96)^**^	0.81 (0.74–0.89)^****^
*≥24 months*	2,238	3.90 ± 1.53	246.22 ± 172.81	101.55 ± 86.95	0.63 (0.54–0.73)^****^	0.59 (0.47–0.72)^****^
**Men**						
*Non-RT*	38,696	0.00 ± 0.00	0.00 ± 0.00	0.00 ± 0.00	1 (reference)^a^	1 (reference)^a^
*<12 months*	916	3.46 ± 1.61	185.28 ± 162.98	5.32 ± 3.02	0.83 (0.70–0.98)^*^	0.92 (0.72–1.19)
*12–23 months*	4,459	4.24 ± 1.83	238.89 ± 196.05	12.02 ± 0.35	0.72 (0.66–0.78)^****^	0.80 (0.71–0.91)^***^
*≥24 months*	1,005	4.02 ± 1.64	247.58 ± 184.12	121.34 ± 105.49	0.52 (0.43–0.62)^****^	0.57 (0.43–0.75)^****^
**Women**						
*Non-RT*	73,523	0.00 ± 0.00	0.00 ± 0.00	0.00 ± 0.00	1 (reference) ^a^	1 (reference) ^a^
*<12 months*	1,470	3.56 ± 1.52	219.29 ± 173.61	4.70 ± 2.88	0.75 (0.60–0.94)^*^	0.78 (0.58–1.04)
*12–23 months*	5,037	4.02 ± 1.62	257.08 ± 187.11	12.05 ± 0.55	0.84 (0.74–0.94)^**^	0.82 (0.71–0.95)^**^
*≥24 months*	1,233	3.81 ± 1.43	245.10 ± 163.08	85.42 ± 63.85	0.58 (0.44–0.77)^***^	0.60 (0.43–0.83)^**^

*RT* resistance training, *OR* odds ratio, *CI* confidence interval, *BMI* body mass index

^a^ *p* < 0.0001 in the test for trend of ORs, ^*^ *p* < 0.05, ^**^ *p* < 0.01, ^***^ *p* < 0.001, ^****^ *p* < 0.0001. Adjusted for age, sex, drinking, smoking, educational level, BMI, hypertension, and diabetes mellitus

**Fig. 2:**
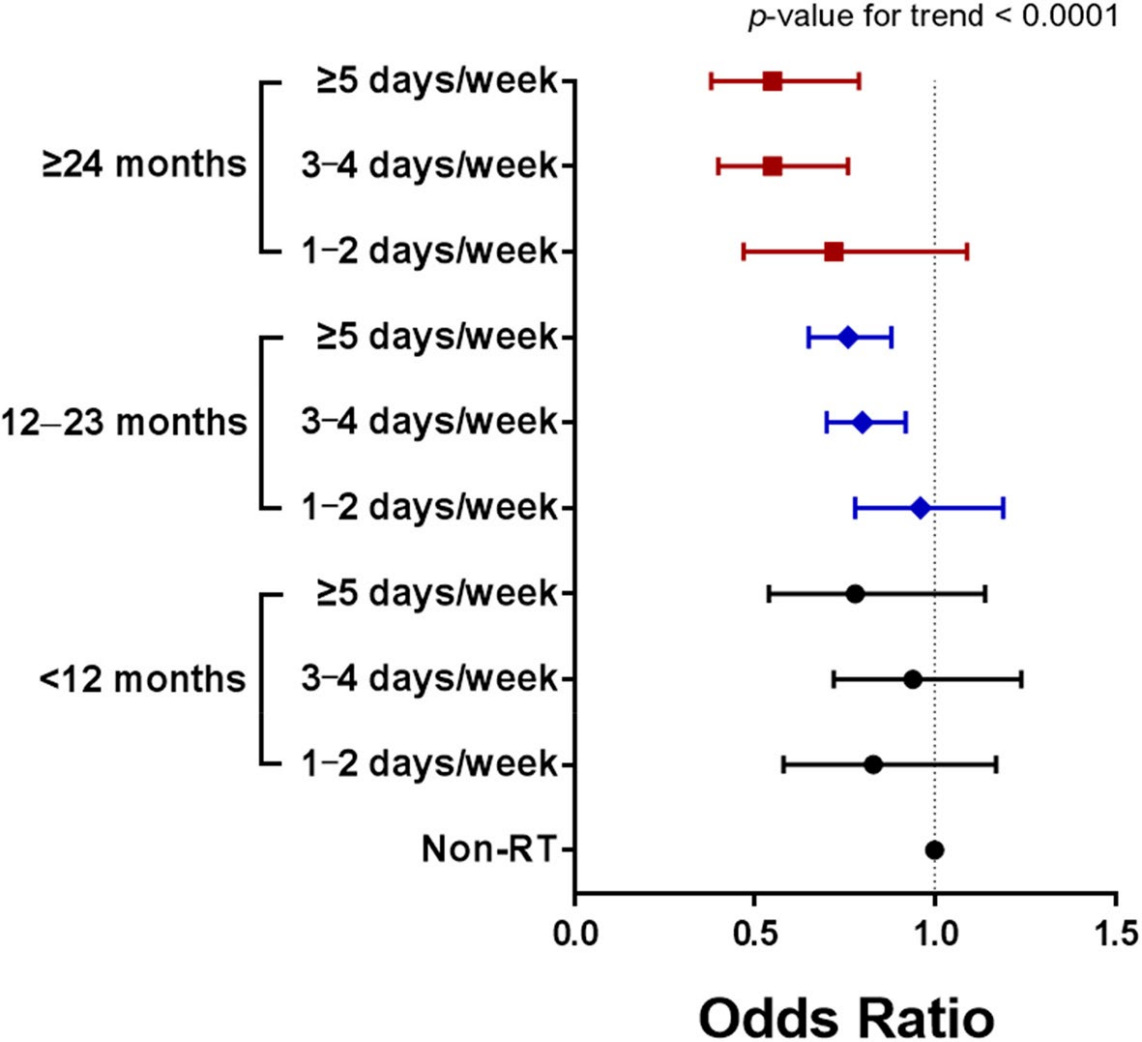
Odds ratios for low muscle mass prevalence according to training period and frequency of RT. Adjusted for age, sex, drinking, smoking, educational level, BMI, hypertension, and diabetes mellitus. RT, resistance training; BMI, body mass index

Figure 2 presents an analysis of the risk of low muscle mass, considering both the training period and frequency of RT after adjustment for covariates. When compared to the Non-RT group, performing RT for 1–2 days/week did not show a significant association with a risk reduction in low muscle mass, regardless of whether RT was performed for more than 1 year. Among participants who performed RT for 1–2 years, performing RT for 3–4 days/week and ≥5 days/week was associated with a risk reduction of 20% (*p* < 0.01) and 24% (*p* < 0.001), respectively, compared to the Non-RT group. Among individuals who performed RT for more than 2 years, compared to those in the Non-RT group, performing RT for 3–4 days/week and ≥5 days/week was associated with a risk reduction of 45% (*p* < 0.001) and 45% (*p* < 0.01), respectively. However, there was no significant difference in the risk of low muscle mass between the 3–4 days/week and ≥5 days/week groups, regardless of whether RT was performed for 1–2 years or more than 2 years.

A subgroup analysis was performed to investigate whether the relationship between the risk reduction of low muscle mass and the performance of RT for ≥3 days/week for more than 1 year was consistently observed in various subgroups, including age, sex, educational level, current drinking habits, smoking status, BMI, hypertension, and diabetes mellitus (Additional file 5). The benefit of performing RT to protect against low muscle mass was consistent across all subgroups except for the BMI subgroup (≥25 kg/m^2^; *p* = 0.05). However, there were no significant interactions observed in the associations across all the subgroups.

## Discussion

One of the major strengths of our study is the use of large nationwide cohorts that are representative of the Korean general population aged 40–79 years. This aspect ensures that our findings can be generalizable to individuals within this age group. Our findings suggest that performing RT for 2 days/week or less is insufficient to prevent low muscle mass. In contrast, performing RT for 3–4 days/week for at least 1 year may confer a protective benefit against low muscle mass, and this benefit can be further enhanced by engaging in long-term RT for more than 2 years. Interestingly, no additional effect was observed when performing RT for 5 days/week or more, regardless of whether it was performed for 1–2 years or more than 2 years. Taken together, we recommend engaging in RT for 3–4 days/week for at least 1 year to prevent low muscle mass.

It is well known that low muscle mass is related to an increased risk of physical dependence, diabetes mellitus, osteoporosis, and all-cause mortality [5–8]. As skeletal muscle mass decreases linearly after the age of 30 years [2], there has been a growing interest in preventive strategies for this harmful condition. For maintaining and/or improving musculoskeletal fitness and health, current guidelines recommend performing RT for 2–3 days per week [12] or at least 2 days per week [18]. In the present study, regular RT significantly reduced the risk of low muscle mass by 22% (Additional file 3), and in participants meeting the current PA guidelines, it further reduced the risk by 24% (Additional file 4), after adjusting for other confounders. Fortunately, the proportion of individuals satisfying the RT recommendations has increased from 17.7% to 27.6% over the past two decades in the United States [13]. However, it remains unclear whether there is an inverse graded dose–response association between RT frequency and the risk of low muscle mass. A previous meta-analysis of RCTs demonstrated that performing RT twice per week was more effective than once per week for increasing muscle mass [14], but additional effects of RT performed at frequencies beyond the current guidelines was not assessed. Our findings suggest that performing RT for 3–4 days/week and ≥5 days/week was associated with a 22% and 27% reduction in the risk of low muscle mass, respectively, after adjusting for covariates, compared to not performing RT. More importantly, despite a significantly higher total training time per week in the ≥5 days/week group compared to the 3–4 days/week group, there was no significant difference in the risk of low muscle mass between these groups. Previous RCTs conducted with healthy young adults demonstrated that when total training volume was equated per week, the 3 days/week group exhibited greater increases in lean body mass and muscular strength compared to the 1 day/week group after 12 weeks of RT [19]. However, no significant differences were observed in fat-free mass and muscular strength between the 3 days/week and 6 days/week groups after 6 weeks of RT [20]. Similar results were obtained even when total training volume per week was not equalized. In an 8-week RT study, the 5 days/week group had a higher total training volume than the 3 days/week group did, but the increases in muscle mass and muscular strength were similar between the groups [21]. Our findings, in conjunction with those from previous studies, indicate that performing RT for ≥5 days/week does not yield additional benefits compared to a frequency of 3–4 times per week, regardless of whether the total training volume per week is equalized or not.

Maintaining muscle mass relies on a fine balance between muscle protein synthesis (MPS) and muscle protein breakdown (MPB). Muscle mass increases when MPS outpaces MPB, while decreases occur when MPB surpasses MPS. Previous studies have shown a robust increase in MPS within the first 24 h after a single bout of resistance exercise [22, 23]. Repeated resistance exercise (i.e., RT) leads to chronic muscle hypertrophy by creating a positive net protein balance [24]. However, when assessed within 24–48 h after a single resistance exercise session, it can also result in delayed onset muscle soreness (DOMS), increased muscle damage (i.e., Z-band streaming area), and increased levels of indirect markers of muscle damage such as creatine kinase and lactate dehydrogenase [24, 25]. Although the magnitude of muscle damage and DOMS gradually decreases with repeated RT sessions, even in untrained participants [26, 27], the magnitude of those was five times higher in the untrained state than in the pre-trained state [28]. Accordingly, post-exercise recovery strategies, such as sufficient rest and adequate consumption of dietary protein, are necessary to maximize post-exercise MPS and facilitate muscle repair. In a previous RCT involving untrained participants, an 8-week moderate-to-high intensity RT program performed at a high training frequency (i.e., 5 days/week) did not lead to greater gains in muscular strength and muscle mass compared to the same RT performed at lower training frequencies, such as 2 days/week and 3 days/week, despite the higher total training volume [21]. Another RCT demonstrated that a 2-week high-intensity RT program performed at a high training frequency (i.e., 6 days/week) resulted in a decline in muscular strength and physical performance, indicating an overtrained state even in trained participants [29]. These findings imply that high training frequencies may impair adequate post-RT recovery between training sessions. Therefore, the current guideline recommends providing ≥48 h of rest between RT sessions to optimize skeletal muscle adaptations [12]. Taken together, our findings, combined with those of previous studies, suggest that performing RT for at least 3–4 days/week with at least 48 h between training sessions is sufficient to prevent low muscle mass by maintaining and/or improving muscular fitness.

It is well documented that during the early phase of RT, there is a rapid increase in muscular strength due to neural adaptations, whereas substantial muscle hypertrophy is only observed after long-term RT programs [30, 31]. Moreover, an initial increase in a post-RT MPS primarily serves for repair and remodeling from muscle damage caused by unaccustomed bouts of RT rather than contributing directly to muscle growth. Notably, substantial muscle hypertrophy is strongly correlated with the summation of post-RT MPS and progressive mitigation of muscle damage during the late phase of a 10-week RT program [24, 32]. A recent meta-analysis of RCTs showed that 8–36 weeks of RT led to a significant increase in muscular strength (e.g., handgrip strength and lower extremity muscle strength) and physical performance but did not improve muscle mass in healthy older adults with sarcopenia [33]. In a previous RCT, high-intensity RT performed for 3 days/week for 6 months significantly increased muscle mass and attenuated muscle mass loss during voluntary weight loss in frail, obese, older adult participants, but the gains in muscle mass were not substantial when compared to baseline measurements [34].

In the present study, which included participants aged 40–79 years, performing RT for <12 months did not correlate with a reduced risk of low muscle mass. However, RT programs lasting 12–23 months and ≥24 months were significantly associated with a reduced risk in both sexes, after adjusting for covariates. Especially, there was a graded dose–response pattern, indicating that longer training periods of RT were linked to greater risk reduction in low muscle mass. These findings are consistent with those from a previous study that conducted a meta-analysis of RCTs involving RT programs lasting 6–52 weeks, which found that the longest training period of RT (i.e., 52 weeks) had the largest effect on both muscle strength and muscle mass in healthy older adults [35].

Interestingly, as shown in Fig. 2 of the present study, performing RT for either 3–4 days/week or ≥5 days/week was significantly associated with a reduced risk of low muscle mass, but only if RT was performed for at least 1 year. On the contrary, performing RT for 2 days/week or less was insufficient for the prevention of low muscle mass, regardless of whether the RT was performed for 1–2 years or more than 2 years. This finding is consistent with previous research results, as high-intensity RT performed for 3 days/week for 1 year has been shown to significantly increase muscular strength, functional performance, lean body mass, and cross-sectional muscle area while decreasing whole-body fat percentage and visceral fat content in older adult participants [36, 37]. Taken together, in order to maintain and/or enhance skeletal muscle mass sufficiently to prevent low muscle mass, it seems necessary to engage in prolonged RT programs with a frequency of 3–4 days per week for at least 1 year. However, the training intensity of prolonged RT, which was not considered in our study, should be evaluated in further studies to clearly suggest the optimal frequency, intensity, type, and training period of RT for the prevention of low muscle mass.

Our study had several limitations. First, the cross-sectional design prevented us from establishing cause-and-effect relationships due to the nature of our study. Second, since our study focused on a Korean population, the generalizability of the findings to other populations may be limited. Third, although the FFMI has been recently validated in the Asian population through measurements of appendicular skeletal muscle mass using both BIA and dual-energy X-ray absorptiometry [17], there is a possibility that the actual prevalence of low muscle mass was either underestimated or overestimated. Fourth, self-reported questionnaires were used to assess RT regularity and leisure-time PA levels, which may have introduced recall bias. Lastly, specific information on RT intensity was not obtained from these self-reported questionnaires. Therefore, further studies are required to determine the optimal frequency, intensity, type, volume, and training period for preventing and/or managing low muscle mass through RT.

## Conclusion

Our findings suggest that following the current RT guidelines, which recommend a minimum frequency of 2 days per week, may be insufficient for reducing the risk of low muscle mass. However, engaging in RT for at least 3–4 days/week for more than 1 year should be considered to prevent muscle mass loss. Therefore, we recommend performing RT for 3–4 days/week for at least 1 year to prevent low muscle mass. The protective benefits can be further enhanced by engaging in long-term RT programs lasting more than 2 years. It is important to note that the present research was a cross-sectional study, and further longitudinal studies are required to validate these findings.

### Supplementary Information

**Additional file 1.** Flow diagram of participant inclusion and exclusion. PA, physical activity.

**Additional file 2.** Characteristics of study participants based on RT regularity and sex.

**Additional file 3.** Odds ratios for low muscle mass prevalence according to RT regularity and sex.

**Additional file 4.** Odds ratios for low muscle mass prevalence according to leisure-time PA-time, RT regularity, and sex.

**Additional file 5.** Odds ratios for low muscle mass prevalence according to RT regularity in various subgroups.

## Data Availability

The data in this study were obtained from the Korean Genome and Epidemiology Study (KoGES; 4851-302), Korea National Institute of Health, Korea Disease Control and Prevention Agency, Korea.

## Abbreviations

ACSM: American College of Sports Medicine
BIA: Bioelectrical impedance analysis
BP: Blood pressure
BMI: Body mass index
CI: Confidence interval
DOMS: Delayed onset muscle soreness
DBP: Diastolic blood pressure
FBG: Fasting blood glucose
FFMI: Fat-free mass index
HDL-C: High-density lipoprotein cholesterol
HEXA: Health examinee
KoGES: Korean Genome and Epidemiology Study
MPB: Muscle protein breakdown
MPS: Muscle protein synthesis
OR: Odds ratio
PA: Physical activity
RCT: Randomized controlled trial
RT: Resistance training
SBP: Systolic blood pressure
T-Chol: Total cholesterol
TG: Triglyceride
WC: Waist circumference

## References

[1] Chen LK, Woo J, Assantachai P, Auyeung TW, Chou MY, Iijima K (2020). Asian working group for sarcopenia: 2019 consensus update on sarcopenia diagnosis and treatment. J Am Med Dir Assoc.

[2] Melton LJ, Khosla S, Crowson CS, O’Connor MK, O’Fallon WM, Riggs BL (2000). Epidemiology of sarcopenia. J Am Geriatr Soc.

[3] Yuki A, Ando F, Otsuka R, Matsui Y, Harada A, Shimokata H (2015). Epidemiology of sarcopenia in elderly Japanese. J Phys Fitness Sports Med.

[4] Kim M, Kim J, Won CW (2018). Association between involuntary weight loss with low muscle mass and health-related quality of life in community-dwelling older adults: nationwide surveys (KNHANES 2008–2011). Exp Gerontol.

[5] Son JW, Lee SS, Kim SR, Yoo SJ, Cha BY, Son HY (2017). Low muscle mass and risk of type 2 diabetes in middle-aged and older adults: findings from the KoGES. Diabetologia.

[6] Kim S, Won CW, Kim BS, Choi HR, Moon MY (2014). The association between the low muscle mass and osteoporosis in elderly Korean people. J Korean Med Sci.

[7] Dos Santos L, Cyrino ES, Antunes M, Santos DA, Sardinha LB (2017). Sarcopenia and physical independence in older adults: the independent and synergic role of muscle mass and muscle function. J Cachexia Sarcopenia Muscle.

[8] Camargo Pereira C, Pagotto V, de Oliveira C, Silveira EA (2022). Low muscle mass and mortality risk later in life: a 10-year follow-up study. PLoS One..

[9] Park JH, Oh J, Park S. Effects of resistance training and/or protein supplementation on usual gait speed in postmenopausal women: a systematic review and meta-analysis. Exerc Sci. 2022;31:26-41. 10.15857/ksep.2022.00024.

[10] Kim KM, Kang HJ. Effects of resistance exercise on muscle mass, strength, and physical performances in elderly with diagnosed sarcopenia: a systematic review and meta-analysis. Exerc Sci. 2020;29:109-20. 10.15857/ksep.2020.29.2.109.

[11] Khodadad Kashi S, Mirzazadeh ZS, Saatchian V (2023). A systematic review and meta-analysis of resistance training on quality of life, depression, muscle strength, and functional exercise capacity in older adults aged 60 years or more. Biol Res Nurs.

[12] Garber CE, Blissmer B, Deschenes MR, Franklin BA, Lamonte MJ, Lee IM (2011). Quantity and quality of exercise for developing and maintaining cardiorespiratory, musculoskeletal, and neuromotor fitness in apparently healthy adults: guidance for prescribing exercise. Med Sci Sports Exerc.

[13] Hyde ET, Whitfield GP, Omura JD, Fulton JE, Carlson SA (2021). Trends in meeting the physical activity guidelines: muscle-strengthening alone and combined with aerobic activity, United States, 1998–2018. J Phys Act Health.

[14] Schoenfeld BJ, Ogborn D, Krieger JW (2016). Effects of resistance training frequency on measures of muscle hypertrophy: a systematic review and meta-analysis. Sports Med.

[15] Kim Y, Han BG, KoGES group. Cohort profile: the Korean Genome and Epidemiology Study (KoGES) consortium. Int J Epidemiol. 2017;46:e20. 10.1093/ije/dyv316. 10.1093/ije/dyv316 PMC5837648 27085081

[16] World Health Organization (2010). Global recommendations on physical activity for health.

[17] Kawakami R, Tanisawa K, Ito T, Usui C, Miyachi M, Torii S (2022). Fat-free mass index as a surrogate marker of appendicular skeletal muscle mass index for low muscle mass screening in sarcopenia. J Am Med Dir Assoc.

[18] Piercy KL, Troiano RP, Ballard RM, Carlson SA, Fulton JE, Galuska DA (2018). The physical activity guidelines for Americans. JAMA.

[19] McLester JR, Bishop P, Guilliams ME (2000). Comparison of 1 day and 3 days per week of equal-volume resistance training in experienced subjects. J Strength Cond Res.

[20] Colquhoun RJ, Gai CM, Aguilar D, Bove D, Dolan J, Vargas A (2018). Training volume, not frequency, indicative of maximal strength adaptations to resistance training. J Strength Cond Res.

[21] Barcelos C, Damas F, Nóbrega SR, Ugrinowitsch C, Lixandrão ME, Marcelino Eder Dos Santos L (2018). High-frequency resistance training does not promote greater muscular adaptations compared to low frequencies in young untrained men. Eur J Sport Sci.

[22] Miller BF, Olesen JL, Hansen M, Døssing S, Crameri RM, Welling RJ (2005). Coordinated collagen and muscle protein synthesis in human patella tendon and quadriceps muscle after exercise. J Physiol.

[23] Phillips SM, Tipton KD, Aarsland A, Wolf SE, Wolfe RR (1997). Mixed muscle protein synthesis and breakdown after resistance exercise in humans. Am J Physiol.

[24] Damas F, Phillips SM, Libardi CA, Vechin FC, Lixandrão ME, Jannig PR (2016). Resistance training-induced changes in integrated myofibrillar protein synthesis are related to hypertrophy only after attenuation of muscle damage. J Physiol.

[25] Barquilha G, Silvestre JC, Motoyama YL, Azevedo PH. Single resistance training session leads to muscle damage without isometric strength decrease. J Hum Sport Exerc. 2018;13:267-75. 10.14198/jhse.2018.132.02.

[26] Chen TC, Yang TJ, Huang MJ, Wang HS, Tseng KW, Chen HL (2019). Damage and the repeated bout effect of arm, leg, and trunk muscles induced by eccentric resistance exercises. Scand J Med Sci Sports..

[27] Nikolaidis MG, Kyparos A, Spanou C, Paschalis V, Theodorou AA, Panayiotou G (2013). Aging is not a barrier to muscle and redox adaptations: applying the repeated eccentric exercise model. Exp Gerontol.

[28] Flann KL, LaStayo PC, McClain DA, Hazel M, Lindstedt SL (2011). Muscle damage and muscle remodeling: no pain, no gain?. J Exp Biol.

[29] Fry AC, Kraemer WJ, van Borselen F, Lynch JM, Marsit JL, Roy EP (1994). Performance decrements with high-intensity resistance exercise overtraining. Med Sci Sports Exerc.

[30] Pearcey GEP, Alizedah S, Power KE, Button DC (2021). Chronic resistance training: is it time to rethink the time course of neural contributions to strength gain?. Eur J Appl Physiol.

[31] Sale DG (1988). Neural adaptation to resistance training. Med Sci Sports Exerc.

[32] Damas F, Libardi CA, Ugrinowitsch C (2018). The development of skeletal muscle hypertrophy through resistance training: the role of muscle damage and muscle protein synthesis. Eur J Appl Physiol.

[33] Chen N, He X, Feng Y, Ainsworth BE, Liu Y (2021). Effects of resistance training in healthy older people with sarcopenia: a systematic review and meta-analysis of randomized controlled trials. Eur Rev Aging Phys Act.

[34] Frimel TN, Sinacore DR, Villareal DT (2008). Exercise attenuates the weight-loss-induced reduction in muscle mass in frail obese older adults. Med Sci Sports Exerc.

[35] Borde R, Hortobágyi T, Granacher U (2015). Dose–response relationships of resistance training in healthy old adults: a systematic review and meta-analysis. Sports Med.

[36] Gylling AT, Eriksen CS, Garde E, Wimmelmann CL, Reislev NL, Bieler T (2020). The influence of prolonged strength training upon muscle and fat in healthy and chronically diseased older adults. Exp Gerontol.

[37] Sundstrup E, Jakobsen MD, Andersen LL, Andersen TR, Randers MB, Helge JW (2016). Positive effects of 1-year football and strength training on mechanical muscle function and functional capacity in elderly men. Eur J Appl Physiol.

