# Supplementary figures and images for: Associations of resistance training levels with low muscle mass: a nationwide cross-sectional study in Korea
Source: Eur Rev Aging Phys Act. 2024 Mar 7;21:5. doi: 10.1186/s11556-024-00339-6 (PMC10918971; doi:10.1186/s11556-024-00339-6)

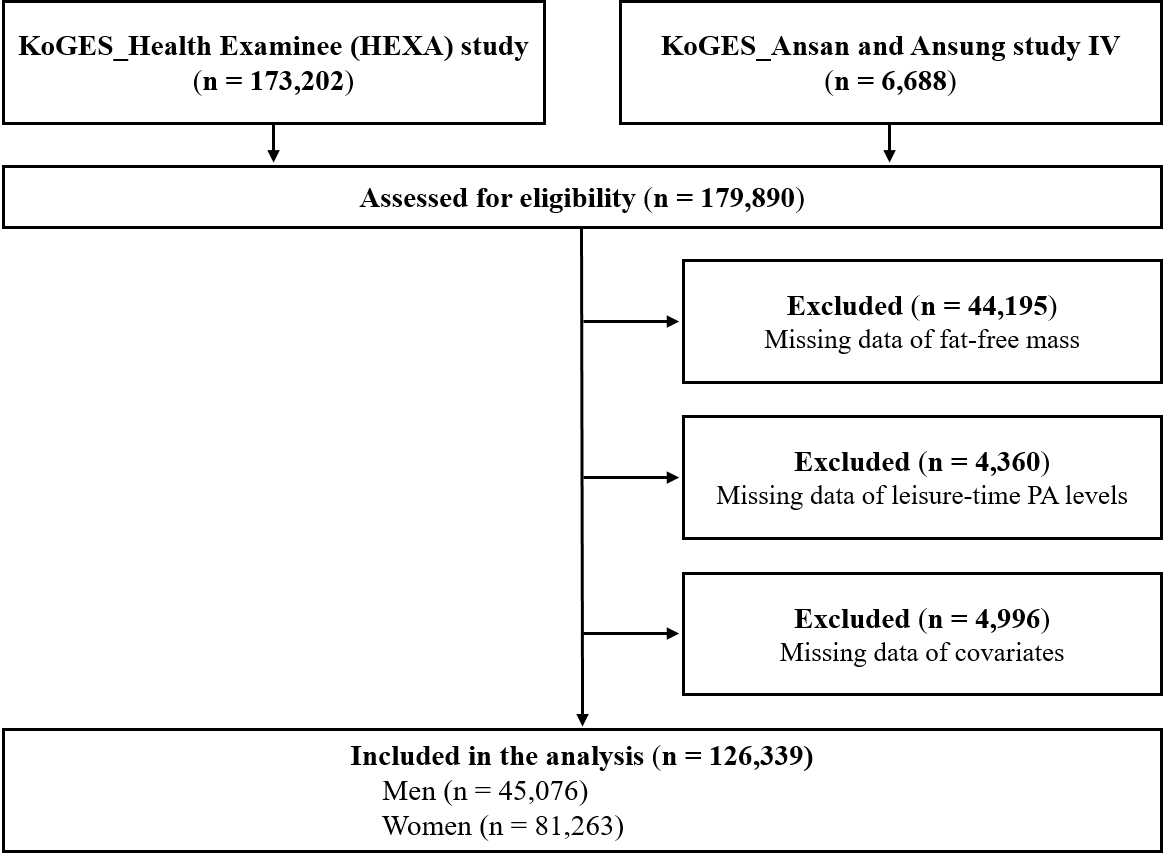


**Additional File 1.** Flow diagram of participant inclusion and exclusion. PA, physical activity.

Supplement: Supplementary file 1 — Additional file 1. Flow diagram of participant inclusion and exclusion. PA, physical activity. [file 11556_2024_339_MOESM1_ESM.doc]
