# Supplementary material for: Associations of resistance training levels with low muscle mass: a nationwide cross-sectional study in Korea
Source: Eur Rev Aging Phys Act. 2024 Mar 7;21:5. doi: 10.1186/s11556-024-00339-6 (PMC10918971; doi:10.1186/s11556-024-00339-6)
Supplement: Supplementary file 2 — Additional file 2. Characteristics of study participants based on RT regularity and sex. [file 11556_2024_339_MOESM2_ESM.doc]

**Additional File 2.** Characteristics of study participants based on RT regularity and sex

| **Variables** | **Men** (n = 45,076) | | ***p*-value** | **Women** (n = 81,263) | | ***p*-value** |
| --- | --- | --- | --- | --- | --- | --- |
|  | **Non-RT**  (n = 38,696) | **RT**  (n = 6,380) |  | **Non-RT**  (n = 73,523) | **RT**  (n = 7,740) |  |
| **Age** (years) | 54.19 ± 8.85 | 53.48 ± 8.48 | < .0001 | 53.18 ± 8.25 | 51.18 ± 7.33 | < .0001 |
| **Educational level**, n (%) |  |  | < .0001 |  |  | < .0001 |
| *≤Elementary school* | 4,027 (10.41) | 261 (4.09) |  | 15,930 (21.67) | 681 (8.80) |  |
| *Middle/high school* | 21,151 (54.66) | 3,050 (47.81) |  | 43,903 (59.71) | 4,867 (62.88) |  |
| *≥College* | 13,518 (34.93) | 3,069 (48.10) |  | 13,690 (18.62) | 2,192 (28.32) |  |
| **Drinking habit**, n (%) |  |  | < .0001 |  |  | < .0001 |
| *Never drinker* | 7,930 (20.49) | 1,104 (17.30) |  | 50,134 (68.19) | 4,774 (61.68) |  |
| *Ex-drinker* | 2,761 (7.14) | 458 (7.18) |  | 1,304 (1.77) | 162 (2.09) |  |
| *Current drinker* | 28,005 (72.37) | 4,818 (75.52) |  | 22,085 (30.04) | 2,804 (36.23) |  |
| **Smoking habit**, n (%) |  |  | < .0001 |  |  | < .01 |
| *Never smoker* | 10,176 (26.30) | 1,811 (28.39) |  | 70,781 (96.27) | 7,462 (96.41) |  |
| *Ex-smoker* | 15,396 (39.79) | 3,006 (47.12) |  | 914 (1.24) | 123 (1.59) |  |
| *Current smoker* | 13,124 (33.91) | 1,563 (24.50) |  | 1,828 (2.49) | 155 (2.00) |  |
| **PA-time** (min/week) | 162.00 ± 254.35 | 315.26 ± 271.35 | < .0001 | 128.65 ± 210.23 | 309.02 ± 243.76 | < .0001 |
| **BMI** (kg/m^2^) | 24.35 ± 2.80 | 24.59 ± 2.59 | < .0001 | 23.71 ± 3.02 | 23.41 ± 2.74 | < .0001 |
| **WC** (cm) | 85.80 ± 7.66 | 85.20 ± 7.23 | < .0001 | 78.78 ± 8.44 | 77.08 ± 7.72 | < .0001 |
| **Fat-free mass** (kg) | 52.94 ± 5.78 | 53.93 ± 5.48 | < .0001 | 39.72 ± 4.01 | 40.26 ± 3.74 | < .0001 |
| **FFMI** (kg/m^2^) | 18.58 ± 1.50 | 18.80 ± 1.42 | < .0001 | 16.26 ± 1.28 | 16.22 ± 1.16 | < .01 |
| **Low muscle mass**, n (%) | 8,531 (22.05) | 1,055 (16.54) | < .0001 | 5,190 (7.06) | 432 (5.58) | < .0001 |
| **SBP** (mmHg) | 124.82 ± 14.43 | 125.32 ± 14.23 | < .05 | 120.73 ± 15.18 | 118.67 ± 14.64 | < .0001 |
| **DBP** (mmHg) | 78.32 ± 9.72 | 78.77 ± 9.75 | < .001 | 74.69 ± 9.68 | 73.76 ± 9.45 | < .0001 |
| **T-Chol** (mg/dL) | 192.59 ± 35.08 | 192.41 ± 33.36 | .70 | 199.17 ± 35.67 | 197.52 ± 34.84 | < .0001 |
| **HDL-C** (mg/dL) | 48.49 ± 11.85 | 49.93 ± 11.83 | < .0001 | 55.09 ± 12.87 | 58.04 ± 13.40 | < .0001 |
| **TG** (mg/dL) | 151.54 ± 108.35 | 143.53 ± 101.32 | < .0001 | 115.10 ± 75.37 | 103.34 ± 63.22 | < .0001 |
| **FBG** (mg/dL) | 99.66 ± 24.99 | 97.88 ± 20.60 | < .0001 | 93.30 ± 19.72 | 91.41 ± 15.87 | < .0001 |
| **Hypertension**, n (%) | 13,575 (35.08) | 2,310 (36.21) | .08 | 19,505 (26.53) | 1,621 (20.94) | < .0001 |
| **Diabetes mellitus**, n (%) | 5,293 (13.68) | 797 (12.49) | < .05 | 6,057 (8.24) | 473 (6.11) | < .0001 |

RT, resistance training; PA-time, total time of regular participation in any sport or exercise to the point of sweating; BMI, body mass index; WC, waist circumference; FFMI, fat-free mass index; SBP, systolic blood pressure; DBP, diastolic blood pressure; T-Chol, total cholesterol; HDL-C, high-density lipoprotein cholesterol; TG, triglycerides; FBG, fasting blood glucose.
