# Supplementary material for: Associations of resistance training levels with low muscle mass: a nationwide cross-sectional study in Korea
Source: Eur Rev Aging Phys Act. 2024 Mar 7;21:5. doi: 10.1186/s11556-024-00339-6 (PMC10918971; doi:10.1186/s11556-024-00339-6)
Supplement: Supplementary file 3 — Additional file 3. Odds ratios for low muscle mass prevalence according to RT regularity and sex. [file 11556_2024_339_MOESM3_ESM.doc]

**Additional File 3.** Odds ratios for low muscle mass prevalence according to RT regularity and sex

|  | **N** | **RT levels** | | | | **Crude model**  OR (95% CI) | **Adjusted model**  OR (95% CI) |
| --- | --- | --- | --- | --- | --- | --- | --- |
|  |  | **Frequency** | **Time** | **Training period** | |  |  |
|  |  | (days/week) | (min/week) | (months) | ≥1 year (%) |  |  |
| **Total** |  |  |  |  |  |  |  |
| *Non-RT* | 112,219 | - | - | - | - | 1 (reference) | 1 (reference) |
| *RT* | 14,120 | 3.99 ± 1.68 | 241.02 ± 185.90 | 25.03 ± 48.05 | 83.10 | 0.85 (0.80–0.89)^****^ | 0.78 (0.72–0.85)^****^ |
| **Men** |  |  |  |  |  |  |  |
| *Non-RT* | 38,696 | - | - | - | - | 1 (reference) | 1 (reference) |
| *RT* | 6,380 | 4.09 ± 1.79 ^a^ | 232.56 ± 190.76 ^a^ | 28.28 ± 58.12 ^a^ | 85.64 ^a^ | 0.70 (0.65–0.75)^****^ | 0.78 (0.70–0.87)^****^ |
| **Women** |  |  |  |  |  |  |  |
| *Non-RT* | 73,523 | - | - | - | - | 1 (reference) | 1 (reference) |
| *RT* | 7,740 | 3.90 ± 1.59 ^a^ | 248.00 ± 181.52 ^a^ | 22.34 ± 37.59 ^a^ | 81.01 ^a^ | 0.78 (0.70–0.86)^****^ | 0.78 (0.68–0.88)^****^ |

RT, resistance training; OR, odds ratio; CI, confidence interval; BMI, body mass index. ^a^ *p* < 0.0001 compared to women with men in the RT group, ^****^ *p* < 0.0001. Adjusted for age, sex, drinking, smoking, educational level, BMI, hypertension, and diabetes mellitus.
