# Supplementary material for: Associations of resistance training levels with low muscle mass: a nationwide cross-sectional study in Korea
Source: Eur Rev Aging Phys Act. 2024 Mar 7;21:5. doi: 10.1186/s11556-024-00339-6 (PMC10918971; doi:10.1186/s11556-024-00339-6)
Supplement: Supplementary file 4 — Additional file 4. Odds ratios for low muscle mass prevalence according to leisure-time PA-time, RT regularity, and sex. [file 11556_2024_339_MOESM4_ESM.doc]

**Additional File 4.** Odds ratios for low muscle mass prevalence according to leisure-time PA-time, RT regularity, and sex

|  | **N** | **RT levels** | | | | **Adjusted model**  **OR** (95% CI) | |
| --- | --- | --- | --- | --- | --- | --- | --- |
|  |  | **Frequency** | **Time** | **Training period** | |  |  |
|  |  | (days/week) | (min/week) | (months) | ≥1 year (%) |  |  |
| **Total** |  |  |  |  |  |  |  |
| *Low-PA* | 73,924 | - | - | - | - | 1 (reference) ^a^ |  |
| *Low-PA+RT* | 3,822 | 3.23 ± 1.72 | 143.70 ± 137.56 | 18.49 ± 36.90 | 73.36 | 0.94 (0.82–1.09) |  |
| *High-PA* | 38,295 | - | - | - | - | 0.91 (0.86–0.97)^**^ | 1 (reference) |
| *High-PA+RT* | 10,298 | 4.27 ± 1.58 | 277.14 ± 188.54 | 27.45 ± 51.37 | 86.72 | 0.69 (0.62–0.76)^****^ | 0.76 (0.68–0.84)^****^ |
| **Men** |  |  |  |  |  |  |  |
| *Low-PA* | 24,238 | - | - | - | - | 1 (reference) ^a^ |  |
| *Low-PA+RT* | 1,820 | 3.36 ± 1.83 | 140.62 ± 143.29 | 21.04 ± 45.14 | 78.35 | 0.90 (0.75–1.09) |  |
| *High-PA* | 14,458 | - | - | - | - | 0.94 (0.87–1.02) | 1 (reference) |
| *High-PA+RT* | 4,560 | 4.38 ± 1.69 | 269.26 ± 194.94 | 31.17 ± 62.32 | 88.55 | 0.71 (0.63–0.81)^****^ | 0.76 (0.66–0.88)^***^ |
| **Women** |  |  |  |  |  |  |  |
| *Low-PA* | 49,686 | - | - | - | - | 1 (reference) ^a^ |  |
| *Low-PA+RT* | 2,002 | 3.11 ± 1.60 | 146.50 ± 132.11 | 16.18 ± 27.15 | 68.83 | 1.00 (0.80–1.24) |  |
| *High-PA* | 23,837 | - | - | - | - | 0.89 (0.82–0.96)^**^ | 1 (reference) |
| *High-PA+RT* | 5,738 | 4.17 ± 1.48 | 283.41 ± 183.06 | 24.49 ± 40.39 | 85.26 | 0.66 (0.57–0.77)^****^ | 0.76 (0.64–0.89)^***^ |

PA-time, total time of regular participation in any sport or exercise to the point of sweating; RT, resistance training; OR, odds ratio; CI, confidence interval; BMI, body mass index. ^a^ *p* < 0.0001 in the test for trend of ORs, ^**^ *p* < 0.01, ^***^ *p* < 0.001, ^****^ *p* < 0.0001. Adjusted for age, sex, drinking, smoking, educational level, BMI, hypertension, and diabetes mellitus.
