# Supplementary material for: Associations of resistance training levels with low muscle mass: a nationwide cross-sectional study in Korea
Source: Eur Rev Aging Phys Act. 2024 Mar 7;21:5. doi: 10.1186/s11556-024-00339-6 (PMC10918971; doi:10.1186/s11556-024-00339-6)
Supplement: Supplementary file 5 — Additional file 5. Odds ratios for low muscle mass prevalence according to RT regularity in various subgroups. [file 11556_2024_339_MOESM5_ESM.doc]

**Additional File 5.** Odds ratios for low muscle mass prevalence according to RT regularity in various subgroups

| **Subgroups** | **N** | **Low muscle mass**,  n (%) | **RT regularity** | | **OR** (95% CI)  Non-RT vs.  ≥3 days/week & ≥1 years | ***p* for interaction** |
| --- | --- | --- | --- | --- | --- | --- |
|  |  |  | **Low muscle mass,** n (%) | |  |  |
|  |  |  | **Non-RT** | **≥3 days/week & ≥1 years** |  |  |
| **Age** (years) |  |  |  |  |  |  |
| *<65* | 107,272 | 12,017 (11.20) | 11,195 (11.37) | 822 (9.35) | 0.77 (0.70–0.85)^****^ | 0.75 |
| *≥65* | 14,653 | 2,683 (18.31) | 2,526 (18.39) | 157 (17.18) | 0.74 (0.57–0.97)^*^ |  |
| **Sex** |  |  |  |  |  |  |
| *Male* | 43,183 | 9,231 (21.38) | 8,531 (22.05) | 700 (15.60) | 0.73 (0.64–0.82)^****^ | 0.61 |
| *Female* | 78,742 | 5,469 (6.95) | 5,190 (7.06) | 279 (5.35) | 0.76 (0.65–0.89)^***^ |  |
| **Educational level** |  |  |  |  |  |  |
| *≤Middle school* | 40,577 | 3,897 (9.60) | 3,764 (9.73) | 133 (7.00) | 0.75 (0.59–0.96)^*^ | 0.95 |
| *≥High school* | 81,348 | 10,803 (13.28) | 9,957 (13.54) | 846 (10.84) | 0.75 (0.67–0.83)^****^ |  |
| **Current drinking habits** |  |  |  |  |  |  |
| *No* | 66,628 | 6,879 (10.32) | 6,461 (10.40) | 418 (9.29) | 0.79 (0.68–0.91)^**^ | 0.25 |
| *Yes* | 55,297 | 7,821 (14.14) | 7,260 (14.49) | 561 (10.77) | 0.70 (0.62–0.80)^****^ |  |
| **Smoking status** |  |  |  |  |  |  |
| *Never* | 105,831 | 11,256 (10.64) | 10,450 (10.74) | 806 (9.41) | 0.73 (0.66–0.81)^****^ | 0.65 |
| *Ever* | 16,094 | 3,444 (21.40) | 3,271 (21.88) | 173 (15.15) | 0.77 (0.60–0.99)^*^ |  |
| **BMI** (kg/m^2^) |  |  |  |  |  |  |
| *<25* | 81,642 | 14,562 (17.84) | 13,590 (18.10) | 972 (14.86) | 0.65 (0.61–0.71)^****^ | 0.42 |
| *≥25* | 40,283 | 138 (0.34) | 131 (0.35) | 7 (0.22) | 0.47 (0.22–1.01) |  |
| **Hypertension** |  |  |  |  |  |  |
| *No* | 86,056 | 11,410 (13.26) | 10,660 (13.47) | 750 (10.84) | 0.75 (0.67–0.84)^****^ | 0.59 |
| *Yes* | 35,869 | 3,290 (9.17) | 3,061 (9.25) | 229 (8.21) | 0.72 (0.59–0.87)^***^ |  |
| **Diabetes mellitus** |  |  |  |  |  |  |
| *No* | 109,617 | 13,403 (12.23) | 12,517 (12.41) | 886 (10.13) | 0.74 (0.67–0.82)^****^ | 0.84 |
| *Yes* | 12,308 | 1,297 (10.54) | 1,204 (10.61) | 93 (9.71) | 0.73 (0.53–1.00)^*^ |  |

RT, resistance training; OR, odds ratio; CI, confidence interval; BMI, body mass index. ^*^ *p* < 0.05, ^**^ *p* < 0.01, ^***^ *p* < 0.001, ^****^ *p* < 0.0001. Adjusted for age, sex, drinking, smoking, educational level, BMI, hypertension, and diabetes mellitus.
